# The prevalence of functional dyspepsia using Rome III questionnaire among chronic hepatitis C patients

**DOI:** 10.1186/s12876-016-0443-2

**Published:** 2016-03-03

**Authors:** Hala I. Mohamed, Hamdy A. Mokarib, Zienab M. Saad, Wael M. Abd El Ghany

**Affiliations:** Faculty of Medicine, Gastroenterology and Endemic Medicine Department, Minya University, Minya, Egypt

**Keywords:** FD, Hepatitis C, PDS, Rome III

## Abstract

**Background:**

Hepatitis C virus (HCV) is a common chronic infection that is widely associated with symptoms of fatigue and abdominal pain. The aim of the present study was to determine the prevalence of functional dyspepsia (FD) among patients with hepatitis C.

**Methods:**

This study included 252 patients with chronic hepatitis C and 150 healthy volunteers. Clinical and laboratory data were recorded for every patient. All patients and controls were administered a questionnaire of FD according to Rome III criteria.

**Results:**

The percentage of patients with FD was significantly higher in patients with chronic HCV than normal controls (65.9 % vs 28.7 %, respectively). In chronic HCV patients, post prandial distention syndrome (PDS) subtype was the predominant type (86.1 %). The percentage of patients with a high fibrosis score (F2–3) and raised ALT were significantly higher in patients with FD than in patients without FD (*P* < 0.001; P < 0.04; respectively). A multivariate regression analysis revealed a significant association between fibrosis score, BMI and FD

**Conclusion:**

FD is more prevalent in patients with chronic hepatitis C. Obese chronic HCV and those with higher fibrosis scores are more likely to have FD.

## Background

FD is a clinical syndrome defined by unexplained chronic or recurrent upper abdominal symptoms [1]. Symptoms are frequently related to meals and include epigastric pain, early satiety, fullness or bloating, belching, nausea and vomiting. In previous report abdominal pain, nausea, bloating, and early satiety were found to occur more frequently in patients with chronic liver disease compared to healthy controls [2]. According to another study, dyspeptic symptoms without any apparent organic cause were reported by 28/62 patients with cirrhosis [3].

The most widely applied criteria used in previous studies were Rome I and Rome II criteria. Due to the strict criteria in Rome II; new Rome III criteria was introduced. In Rome III criteria two subtypes of FD were identified epigastric pain syndrome (EPS) and post prandial distress syndrome (PDS) [4]. little is known about these subtypes especially in our Egyptian community.

Functional dyspepsia have been studied in patients with hepatitis C [5], but no published data are yet available about the possible association between FD and chronic HCV infection. The aim of this study was to investigate the possible association between chronic hepatitis C and FD.

## Methods

This study was under taken at the Virology and Hepatology clinic of Minia University Hospital, Minia, Egypt. The study was approved by the institutional review board of the Minia Faculty of Medicine, Minya University, Egypt. Written, informed consent was obtained from every participant (for both participation in the study and publication of the data). In total, 252 consecutive patients with chronic hepatitis C genotype 4, diagnosed by HCV-RNA and liver biopsy using the Metavir score, were enrolled in the study. One hundred and fifty controls were randomly selected from healthy volunteer (medical officer, nurses, medical staff and medical students. They were matched in age and sex to the patients. All patients and controls were requested to fill out a self-report questionnaire. The questionnaires were dispensed to patients and controls by the researchers. They were requested to consult the doctor if they did not understand a question in the questionnaire. A researcher assisted those with literacy difficulties.

The validated Rome III diagnostic questionnaire for FD was designed by the Rome committee. The FBDs module was downloaded from the Rome Foundation website (http://www.romecriteria.org/questionnaires/).

FD patients were classified into two subtypes PDS and EPS. PDS must include all of the following: Bothersome postprandial fullness, occurring after ordinary sized meals, at least several times per week. Early satiation that prevents finishing a regular meal, at least several times per week. EPS must include all of the following: 1) Pain or burning localized to the epigastrium, of at least moderate severity at least once per week, 2) The pain is intermittent, 3) Not generalized or localized to other abdominal or chest regions, 4) Not relieved by defecation or passage of flatus, 5) Not fulfilling criteria for biliary pain, 6) Criteria fulfilled for the last 3 months with symptom onset at least 6 months prior to diagnosis..

The alarm (or red flags symptoms) are also included in the questionnaire. The presence of alarm symptoms as melena, hematemesis, loss of weight, history of gastric cancer potentially indicative of organic diseases that warrant further investigations and necessitates for further evaluation.

In addition to that the psychological alarm questionnaire which help to detect associated psychological problems related to FD. The questions include anxiety, depressive symptoms, suicidal ideation, severity of pain, physical impairment and abuse behavior. Patients who fulfilled the criteria for FD based on Romi III questionnaire were referred to endoscopy to rule out the organic cause.

### Statistical analysis

Statistical analysis was applied using Graph pad prism software with curve fitting version 4 and SPSS computer program (version 13 windows). Contingency tables used (chi square and Fisher’s tests) for binomial variables and T test and AVOVA tests for the other variables. Multivariate regression analysis was done. P value equal to or less than 0.05 was considered significant. Results were expressed as mean ± SD unless otherwise stated.

## Results

Total of 272 HCV patients and 150 normal controls patients were studied. Twenty one patients were non responders as they did not complete the questionnaire (10 patients) and refusing endoscopy (11 patients). Of the 251 HCV infected patients were included in the study; 187 fulfilled the criteria of functional dyspepsia and underwent upper endoscopy. One hundred sixty six had normal endoscopy and were diagnosed as having functional dyspepsia, 5 subjects had lower esophagitis, 8 patients had gastro esophageal reflux disease, 6 subjects had antral gastritis and duodenal ulcer recorded in 2 patients

There was significant difference in the demographics between the control groups and the HCV group with regard to age, sex, smoking and BMI (P < 0.001; P < 0.0001; *P* < 0.0001; *P* < 0.003, respectively),other socio-demographics character of the precipitants was shown in (Table 1).

**Table 1:** Socio-demographic and clinical criteria of the patients and controls

Parameter	HCV patients	Normal control	*P*-value
	252	150	
Age (mean ± SD)	45.5 ± 8.08	33.6 ± 12.09	<0.001*
Sex			<0.0001*
Male	161 (63.9 %)	53 (35.3 %)	
Female	91 (36.1 %)	97 (64.7 %)	
Residence			<0.9
Urban	156 (61.9 %)	93 (62 %)	
Rural	96 (38.1 %)	57 (38 %)	
Years of education (Mean ± SD)	9.70 ± 2.9	10.4 ± 1.8	<0.13
Smoking			<0.0001*
Yes	138 (54.8 %)	13 (8.7 %)	
No	114 (45.2 %)	137 (91.3 %)	
BMI (kg/m^2^)	29.4 ± 3.9	23.9 ± 3.5	<0.003*
Red flag symptoms			<0.09
Yes	10 (4 %)	2 (1.3 %)	
No	242 (96 %)	148 (98.7 %)	
Psycho-social symptoms			<0.2
Yes	9 (3.6 %)	12 (8 %)	
No	243 (96.4 %)	138 (92 %)	

*HCV* hepatitis C virus, *BMI* body mass index, *FD* functional dyspepsia

*Statistically significant

**Fig. 1:**
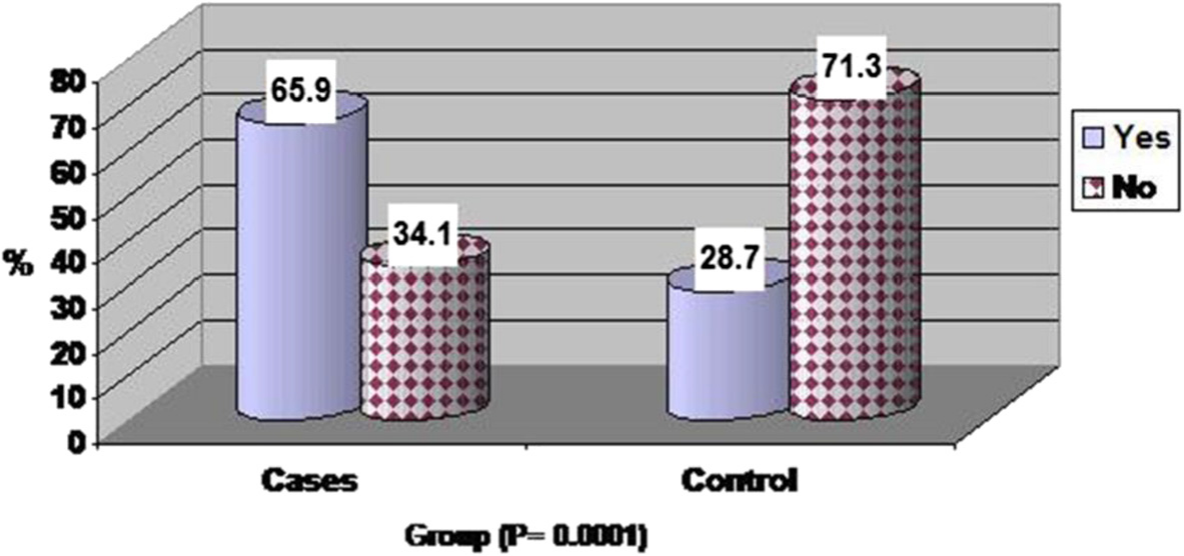
Prevalence of Functional dyspepsia in patients with chronic hepatitis C. Percentage of patients with FD diagnosed according to Rome III criteria was significantly higher in patients with chronic hepatitis C virus (65.9 %) than in controls (28.7 %)

The percentage of patients with FD diagnosed according to Rome III criteria were significantly higher in patients with chronic HCV infection than normal controls (65.9 %, 28.7 %; respectively; *P* < 0.0001) Fig. 1. Regarding to functional dyspepsia subtypes, the PDS subtype was significantly predominant than The EPS one in both patients and controls (*P* < 0.005, *P* < 0.03) (Table 2).

**Table 2:** Prevalence of functional dyspepsia subtypes among HCV patients and controls

Parameter	HCV positive patients	Normal controls
	n = 166	n = 43
Patients with PDS	153 (86.1 %)	28 (65.1 %)
Patients with EPS	13 (13.9 %)	15 (34.9 %)
*P* value	<0.005**	<0.03**

*HCV* Hepatitis C virus; *PDS* Post prandial distress syndrome; *EPS* Epigastric pain syndrome

**Statistically significant

To study the possible risk factors of FD in patients with chronic HCV, we compared patients with FD to those without. It was found that ALT level and fibrosis stages were significantly higher in patients with FD than in patients without (*P* < 0.04, *P* < 0.0001), there was no significant difference in HCV viral load in patients with FD than in patients without (*P* < 0.2) (Table 3).

**Table 3:** Biochemical, serological and pathological characteristics of HCV patients with functional dyspepsia

Parameter	Patients with FD	Patients without FD	*P*-Value
	166	86	
Serum ALT			
Normal	9 (45 %)	11 (55 %)	<0.04*
Elevated	157 (67.7 %)	75 (32.3 %)	
Fibrosis score			<0.0001*
F0-F1	13 (37.1 %)	22 (62.9 %)	
F2-F3	153 (70.5 %)	64 (29.5 %)	
HCV RNA (PCR)			<0.2
Median	147,160	180,603	
Range	(1000–2 220 000)	(800–4 080 000)	

*ALT* alanine transaminase; *FD* functional dyspepsia; *PCR* Polymerase chain reaction

*Statistically significant

Multivariate regression analysis was done to clarify the association between patient’s risk factors and functional dyspepsia. The most important predictors of FD in HCV patients were BMI (*P* < 0.01) and fibrosis stage (*P* < 0.001). There was no significant association between age, sex, smoking, ALT, HCV viraemia and FD (Table 4).

**Table 4:** Multivariate regression analysis of factors associated with FD In HCV patients

Parameter	Adjusted odds ratio (95 % CI)	P-value
Age > 40	1.4 (0.96–1.04)	<0.8
Male Sex	0.74 (0.37–1.46)	<0.3
BMI >25 kg/m^2^	1.61 (1.35–3.16)	<0.01*
Smoking	1.77 (0.87–3.58)	<0.1
High serum ALT	1.2 (0.98–0.01)	<0.8
High viraemia (PCR)	1 (1–1)	<0.2
Fibrosis score (F3-F4)	3.8 (1.76–8.52)	<0.001*

*ALT* alanine transaminase, *FD* functional dyspepsia, *PCR* Polymerase chain reaction; *CI* confidence interval

*statistically significant

## Discussion

Patients with Chronic hepatitis C usually have different abdominal complaints. Abdominal pain or discomfort is frequently seen in clinical practice in patients with chronic HCV without organic lesion, the functional origin of abdominal complaints is claimed in many patients [6].

We have seen this association frequently in clinical practice, but this is the first designed study to investigate the possible association between FD and chronic hepatitis C.

In this study, the prevalence of FD was significantly high in HCV patients than healthy controls (65.9 % vs 28.7 %). It was found that the majority of the HCV infected patients in this study had symptoms of PDS which constituted about 86.1 % of them while 13.9 % of patients had EPS.

This finding was similar to most studies performed as a population based research. Aro et al. [7] in Sweden who found that PDS was more prevalent than EPS. These findings indicate that patients with liver disease have meal-related symptoms that may be under recognized. Further investigational studies of gastric neuromuscular function, such as gastric emptying studies and electrogastrogram testing, are warranted. Intestinal motility disorders have been described in cirrhosis but not previously reported or studied in chronic hepatitis [8].

Alternative explanations for these postprandial symptoms might include meal-related increases to portal blood flow and volume changes. Duplex ultrasound and MRI have shown that patients with chronic hepatitis C have a greater volume change to the left lobe of the liver with a relatively smaller change in portal blood flow than do controls [9].

Theoretically, portal flow and volume changes in patients with inflammatory conditions of the liver may result in more congestion and subsequent stretch of Glisson’s capsule, in turn leading to more stimulation of hepatic vagal afferent activity with resultant symptoms of postprandial pain, nausea, and fullness [10].

Grassi et al., reported FD in 28.8 % of patients with chronic liver diseases and 71.2 % had organic cause of dyspepsia as gastroesophageal reflux disease, congestive gastropathy, gastric or duodenal ulcer and gallbladder stones. Severity of dyspeptic symptoms was similar in both organic and functional forms; symptoms tend to occur with moderate intensity, worsening in parallel with the aggravation of liver disease. The predominance of functional forms in liver diseases is practically the same as that reported in the general population [11].

By multivariate analysis, the most important factors that predicts FD were BMI and fibrosis scores. While gender, age, residence, smoking and viral load were not significantly associated. Aro et al. demonstrated that obesity (BMI > 30 kg/m^2^) was predictive for uninvestigated FD but no significant association between obesity and FD [7].

A higher fibrosis scores in HCV patients with FD can indicate a prolonged duration of HCV infection and associated motor or inflammatory changes in the bowel mucosa. Previous studies indicated that patients with cirrhosis were found to have increased severity of gastrointestinal symptoms correlating with the severity but not the etiology of the liver disease [12]. This means that increasing grade of fibrosis is associated with increase occurrence of FD this may occur as a result of immunological changes that occur with advanced liver diseases.

Gastrointestinal sensorimotor disturbances have previously been reported in patients with cirrhosis and might be involved in the pathogenesis of gastrointestinal symptoms in these patients.

In summary FD is a prevalent finding in HCV patients and its pattern is not different from that of the general population.

## Conclusion

FD is more prevalent in patients with chronic hepatitis C. Obese, chronic HCV patients and those with higher fibrosis scores are more likely to have FD. Further studies are needed to determine why. Is infectious pathogenesis, motor dysfunction, or psychological disturbance?.

Limitation of the study: as this study employed questionnaire method for data collection it might lead to recall bias and language bias. Also some subjects in this study had actually being treated for their dyspeptic symptoms and this would change the course of the symptoms and could affect the true prevalence of FD.

## Abbreviations

HCV: hepatitis C virus
FD: functional dyspepsia
PDS: post prandial distention syndrome
EPS: epigastric pain syndrome
ALT: alanine aminotransferase
BMI: Body Mass Index
95 % CI: 95 % confidence interval
OR: odds ratio
